# High risk of hip dislocation following polyethylene liner exchange in total hip arthroplasty—is cup revision necessary?

**DOI:** 10.1007/s00402-020-03603-3

**Published:** 2020-09-19

**Authors:** D. Dammerer, F. Schneider, T. Renkawitz, D. Putzer, M. Bogensperger, R. Biedermann

**Affiliations:** 1 Department of Orthopaedics and Traumatology, Medical University of Innsbruck, Anichstrasse 35, 6020 Innsbruck, Austria; 2 Department for Trauma Surgery and Sports Medicine, Medical University of Innsbruck, Anichstrasse 35, 6020 Innsbruck, Austria; 3 Department of Orthopedic Surgery, Regensburg University Medical Center, Kaiser Karl V.-Allee 3, 93077 Bad Abbach, Germany; 4 Department of Experimental Orthopedics, Medical University of Innsbruck, Sonnenburgstrasse 16, 6020 Innsbruck, Austria

**Keywords:** Liner exchange, Polyethylene wear, Revision arthroplasty, Dislocation, EBRA

## Abstract

**Purpose:**

Polyethylene (PE) wear remains a common reason for revision surgery following total hip arthroplasty (THA). An established treatment method is isolated liner exchange in a well-fixed acetabular cup and entails a known high risk of hip dislocation after revision surgery. The purpose of this retrospective study was to determine the rate of hip dislocation after liner exchange.

**Methods:**

Patients were included if (1) the PE liner was removable, (2) the acetabular shell was stable with acceptable orientation, (3) no osteolysis around the acetabular cup was found and (4) no dislocation of the THA occurred before revision surgery. We reviewed medical histories and performed radiological measurements using Einzel-Bild-Röntgen-Analyse (EBRA) software. EBRA measurements and statistical investigations were performed by two independent investigators.

**Results:**

A total of 82 patients were included in our study. Mean follow-up was six (range: 3.6–9.9) years. In 13 (15.8%) patients THA dislocations occurred at a mean postoperative period of 20.2 (range: 1–44) weeks after revising the PE liner. This is equivalent to an absolute risk increase of 16% after revision surgery, which results in a number needed to harm of 6. This means that every sixth patient with isolated liner exchange can expect to experience dislocation due to wear.

**Conclusion:**

In conclusion, isolated exchange of the polyethylene liner because of wear showed a high risk of dislocation and further cup revision. Our results suggest that the threshold for revising well-fixed components in the case of liner wear should be lowered.

**Trial Registration number and date of registration:**

Number: 20140710-1012 and Date: 2016-03-09.

## Introduction

Polyethylene (PE) wear remains a common reason for revision surgery following total hip arthroplasty (THA) [1, 2]. The results of different arthroplasty registers show that replacing a PE liner because of wear becomes necessary in approximately 10–20% of arthroplasty cups [3, 4]. PE wear in a well-fixed uncemented cup is often treated by exchanging the PE liner and femoral head [1]. Whether to revise a well-fixed and well-positioned acetabular component in the presence of PE wear and osteolysis remains controversial [1]. Choosing the best procedure for revision surgery in patients showing PE wear involves the difficult decision whether to revise only the liner (e.g. lipped liner, constrained liner, increasing femoral head size) and face a high risk of postoperative hip dislocation or to revise both the cup (e.g. reposition of the cup) and the liner and use a dual-mobility component to reduce the risk of hip dislocation [5].

The current literature reports considerable disagreement over the revision method preferred for revising worn-out liners. Previously published studies argue that leaving the well-fixed and well-positioned cup unchanged at revision diminishes the probability of postoperative dislocation [6–8], whereas other reports claim the opposite [9, 10]. Some authors advocate acetabular cup revision, while others insist on cup preservation with exchange of the liner only [6, 11]. Some studies have shown that leaving a well-fixed acetabular shell in situ may also lead to an increased risk of instability [12].

These considerations need to be balanced against the otherwise low complication rate for revision of the liner [12]. Replacement of a PE liner is considered more benign than full acetabular revision and a relatively simple procedure with less blood loss, shorter operation and hospitalization time [5]. Therefore, isolated exchange of the liner has become an increasingly common indication for revision surgery, even when facing a high rate of dislocation [9].

Among our patients we observed an unexpectedly high incidence of recurrent dislocation after liner exchange followed by revision of the cup. The purpose of this retrospective study was to determine the rate of THA dislocation following liner exchange.

## Materials and methods

The study was approved by the local ethics committee (Medical University of Innsbruck, Austria). We retrospectively investigated all consecutive patients at our department who between January 2000 and December 2014 underwent isolated PE liner exchange for wear with retention of the acetabular cup and femoral stem. Patients were included if (1) the PE liner was removable, (2) the acetabular shell was stable with acceptable orientation, (3) no osteolysis around the acetabular cup was found, and (4) no dislocation of the THA occurred before revision surgery. Exclusion criteria were revision of either the acetabular or the femoral component at the same intervention or a diagnosis of infection.

**Fig. 1:**
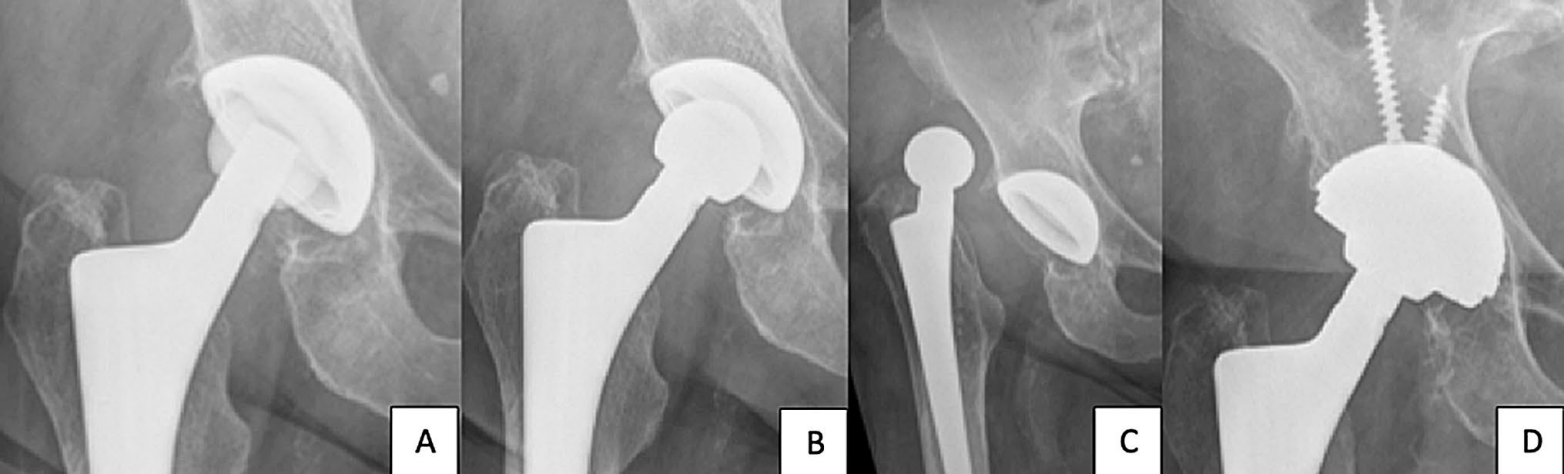
Massiv PE liner wear **a** without osteolysis around the cup or the greater trochanter. Postoperative x-ray control after revision of the liner. **b** Dislocation after liner exchange. **c** Cup revision to a dual mobility liner and cup with additionally screw fixation

**Fig. 2:**
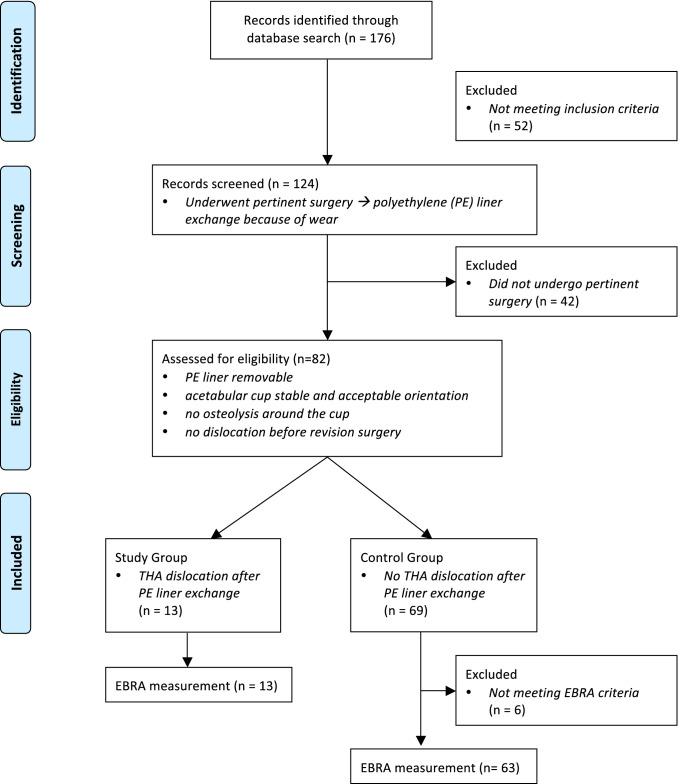
PRISMA 2009 flow diagram detailing inclusion and exclusion of the patients

We identified a total of 124 patients, 82 of whom fulfilled the inclusion criteria. We then divided our study population of 82 patients into two groups (a) liner exchange without postoperative THA dislocation (control group) and (b) liner exchange with postoperative THA dislocation (study group; Fig. 1). Patient flow chart is shown in Fig. 2.

We also investigated patient medical histories for sociodemographic data, surgical approach, cut to suture time, pre- and postoperative range of motion, Charlson Comorbidity Score [13], body mass index, femoral and acetabular offset, details of revision procedure and blood loss during surgery, Estimated blood loss was calculated using the formula of Meunier [14]. Each substitution of concentrated red blood cells, administered intra- or postoperatively until the fifth day, was included in the calculation with a quantity of 280 ml (ml) and a haematocrit of 0.54.

Prosthetic stability, PE wear and cup migration were retrospectively assessed with EBRA (German: Einzel-Bild-Röntgen-Analyse) [15] from plain x rays. EBRA is a well-established method that evaluates standard anterior–posterior radiographs without requiring additional means at exposure (e.g., ball markers). Simulating the spatial situation, it computes parameters of longitudinal and transverse migration of prosthetic cup, femoral head and wear. The migration of the femoral head, the acetabular cup and wear in the horizontal and vertical directions can be studied. Total wear was calculated from the EBRA wear results in the horizontal and vertical directions by vectorial addition to make the results comparable with those of other methods. Furthermore, total wear was calculated as the differences between migration of the head and cup in the horizontal and vertical directions. A comparability algorithm using a grid of transverse and longitudinal tangents of the pelvis contour divides serial radiographs into sets of comparable ones. Migration is measured only between comparable radiographs. The 95% confidence limits for EBRA results are 1.0 mm for longitudinal and 0.8 mm for transverse migration [15].

In our department, we routinely follow patients with radiographs before discharge, 6 weeks after surgery and 12 month postoperative. We perform additional radiographs if the patient has any complaints with the THA. All radiographs were taken at our Department of Radiology with the same technique (anterior–posterior (AP) radiographs; patient standing in upright position and full weight-bearing). For our EBRA investigation, a minimum of three radiographs per patient and a minimum radiological follow-up of up to 6 months was required for this analysis. Cup migration analysis was done with EBRA by one independent investigator, who was not involved in the surgeries or postoperative treatment of patients.

Additionally, to preoperatively rule out an infection, a preoperative fluoroscopy-guided joint fluid sample was gathered (study group: *n* = 8/13; control group: *n* = 38/69). In all cases no infection was found. Unfortunately, no further investigation was performed, e.g., histopathological diagnosis of the synovia for the identification of particles of prosthesis material or polyethylene particles. This would have been of great importance in the evaluation of implant failure. An additional comment will be added in the Limitations section.

### Statistical analysis

Mean, median, range, and standard deviation were calculated for the various measurement parameters. For analysis, Access and Excel (Microsoft Office Professional Plus 2010, Redmond, US-WA) as well as Graph Pad Prism (Version 7.0, GraphPad Software, Inc., La Jolla, US-CA) were used. For pairwise comparison of various parameters the non-parametric Mann–Whitney *U* test was used. Two-Way ANOVA with Sidak correction for multiple comparisons was used to assess statistical significance within migration, wear rates, inclination and anteversion. A *p* value of 0.05 was considered statistically significant.

## Results

The most important findings in our study were the high rate of THA dislocation and the unexpectedly high rate of cup revision after liner exchange in our study group. We found an absolute risk increase of 16% after revision surgery, which results in a number needed to harm of 6. This means that every sixth patient with isolated liner exchange can expect to experience dislocation due to wear.

### Sociodemographics

As mentioned above, we divided our study population into a study (*n* = 13) and a control (*n* = 69) group. The study group included 13 patients (10 female, 3 male) with a mean age of 70 (range: 46–83) years at PE liner exchange. The control group consisted of 69 (37 female, 32 male) patients with a mean age of 67 (range: 26–83) years at time of revision surgery. No significant difference was found between the two groups for comorbidities following the Charlson Comorbidity Score [13] (*p* = 0.2). Details shown in Table 1.

**Table 1:** Sociodemorgaphic data, EBRA cup measurement results, blood loss, surgical approach and used liner at the revision surgery

	Study group	Control group	*p* values
Participants	13 (f. 10; m 3)	69 (f.37; m32)	n.s
Age at revision surgery (yr)	70 (46–83)	67 (26–83)	n.s
Body-mass index	28 (21.4–42.7)	37.7 (17.7–38.7)	n.s
Charlson comorbidity Index	2.8 (0–7)	3.7 (0–13)	n.s
*Surgical approach inital surgery*			
Lateral	13	43	
Direct anterior approach	0	12	
Missing	0	14	
*Revision Surgery – Liner Exchange*			
Surgical approach by revision			
Lateral	2	14	
Direct anterior approach	11	54	
Missing	0	1	
Cut to suture time (min.)	60 (± 21)	60 (± 27)	n.s
Blood loss in ml	800 (± 600)	900 (± 600)	n.s
*Used liner in revision*			
Neutral	9	46	
10° lipped	3	21	
Constrained	1	0	
Missing	0	2	
Radiological follow up (yr.)	6 (3.6–9.9)	6 (3.6–9.9)	n.s
*EBRA cup measurments*			
Medial migration (mm)	3 ( – 2–7.9)	1.5 ( – 2.6–1.6)	0.032
Cranial migration (mm)	2.6 ( – 1.4–2.5)	2 ( – 1.8–28.9)	0.001
Inclination	45.5° (25.4–56.7)	44° (33.2–55.9)	n.s
Anterversion	16.4° (7.2–23.4)	18.2° (6.5–34.6)	n.s
Wear (mm)	0.3 (0–3.7)	0.15 ( – 2.6–1.6)	n.s

*F* female, *m* male, *yr* years, *min* minutes, *mm* millimetre, *n.s.* not significant

### Implant survival and revision surgery indication

In our study group (*n* = 13), revision surgery because of PE wear was necessary at a mean of 10.8 (range: 8.3–13.5) years after primary THA, while the control group (*n* = 69) underwent liner exchange after a mean of 9.5 (range: 0–16.7) years. No statistically significant difference in implant survival rates (*p* = 0.5) was found between the two groups. The indication for exchange of the liner in both groups was PE wear. In the study group, all 13 patients had a DePuy Synthes (DePuy-Synthes, Warsaw IN, USA) Duraloc® Marathon neutral liner inserted, while in the control group 44 patients received a DePuy Synthes (DePuy-Synthes, Warsaw IN, USA) Duraloc® Marathon liner (neutral) and 25 a Stryker (Stryker Howmedica Osteonics) Crossfire® liner (neutral). After revising the PE liner, an average of three (range: 1–6) THA dislocation occurred after a mean of 20.2 (range: 1–44) weeks postoperative. In the control group no postoperative dislocation occurred. All THA dislocations were repositioned under fluoroscopic guidance. After a mean of one (range: 0–2) years and recurrent THA dislocations six patients (*n* = 6/13) in the study group underwent cup replacement to a dual-mobility cup, three patients (*n* = 3/13) received a 10° lipped liner and four patients (*n* = 4/13) a constrained liner. After the second revision surgery no dislocation was noticed in our patient reporting system in the investigated follow-up period.

### Surgical approach

All patients in the study group (*n *= 13) were initially operated through a lateral-transgluteal approach [16]. At liner revision the approach was converted in 11 patients (*n* = 11/13) to a direct anterior approach (DAA) [17, 18], while in two patients (*n* = 2/13) the initial lateral-transgluteal approach was used. In the control group 12 patients (*n* = 12/69) were initially operated with a DAA and 43 patients (*n* = 43/69) with a lateral-transgluteal approach. Information on the surgical approach is missing in 14 cases (*n* = 14/69). Liner revision surgery was performed with a DAA in 54 (*n* = 54/69) cases, while in 14 patients (*n* = 14/69) the lateral-transgluteal approach was used. In one patient the approach for revision surgery was not recorded (*n* = 1/69).

### Head and Liner

In the control group, PE liner revision surgery was performed in 46 patients (*n *= 46/69) with a neutral liner and in 21 patients (*n* = 21/69) with a 10° lipped liner. Information on the used liner is missing in two patients (*n* = 2/69). In the study group, nine patients (*n* = 9/13) received a neutral liner, three patients (*n* = 3/13) a 10° lipped PE liner and one patient (n = 1/13) a constrained liner. Head size was changed from 28 mm (mm) to 32 mm in five patients (*n* = 5/13), in an additional five patients (*n* = 5/13) head size was not changed (32 mm) and in three patients (*n* = 3/13) in the study group this information is missing. In the control group, head size was changed from 28 to 32 mm in 23 patients (*n* = 23/69), in 12 patients (*n* = 12/69) head size was not changed (32 mm) and in 34 patients (*n* = 34/69) this information is missing. Expect the missing data, in all other cases the head length was not changed during revision surgery.

### EBRA measurements

**Fig. 3:**
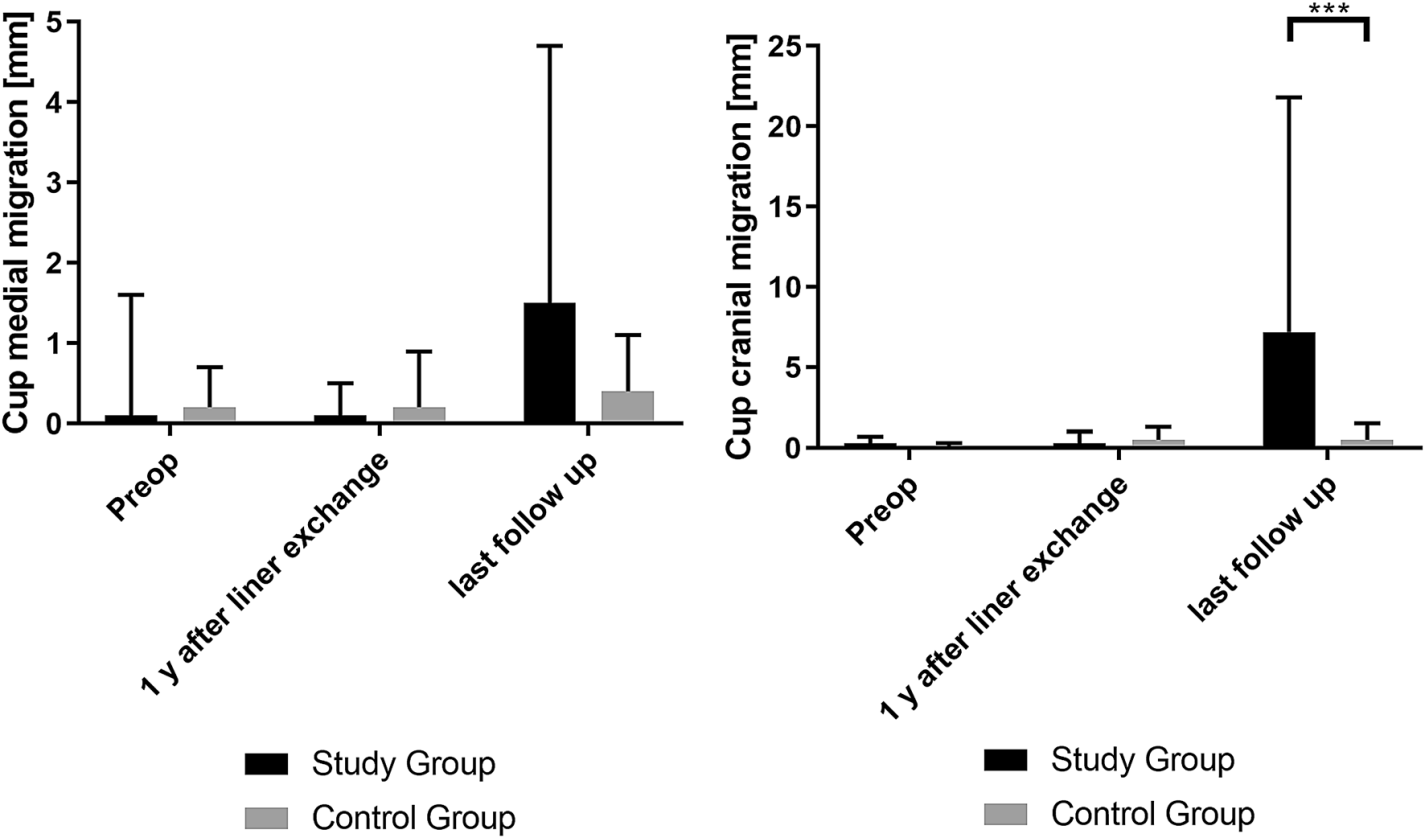
Mean cup migration and standard deviation of EBRA-cup analysis. Medial and cranial cup migration in the study group at last follow-up after liner exchange was significantly increased

Mean radiological follow-up was six (range: 3.6–9.9) years for both groups. All patients in the study group fulfilled the EBRA criteria, while six patients in the control group had to be excluded due to incomplete radiologic follow-up. EBRA analysis showed a significant difference between the two groups in medial (*p* = 0.032) and cranial cup migration (*p* = 0.001). Mean medial cup migration in the study group was 3 mm (range:  – 2 to 7.9), while in the control group 1.5 mm (range:  – 2.6 to 1.6). Mean cranial cup migration was 2 mm (range:  – 1.8 to 28.9) in the study group and 2.6 mm (range:  – 1.4 to 2.5) in the control group. Details are shown in Fig. 3. Wear measurements with EBRA did not show a statistically significant difference (*p* = 0.968) between both groups. Mean total wear in the study group was 0.3 mm (range: 0–3.7) and 0.15 mm (range:  – 2.6 to 1.6) in the control group.

No significant difference was found for the cup inclination (*p* = 0.166) and anteversion (*p* = 0.234) between the two groups. Mean inclination in the study group was 45.5° (range: 25.4°–56.7°) and in the control group 44° (range: 33.2°–55.9°). Mean cup anteversion in the was 16.4° (range: 7.2°–23.4°) for the study group and 18.2° (range: 6.5°–34.6°) for the control group. In addition, no significant difference in postoperative femoral (*p* = 0.196) or acetabular offset was observed between the two groups (*p* = 0.702).

## Discussion

The purpose of the present study was to determine the rate of THA dislocation after liner exchange because of PE wear in a well-fixed and orientated cup without osteolysis. The most important findings in our study were the high rate of THA dislocation and the unexpectedly high rate of cup revision after liner exchange in our study group.

According to the literature, treating polyethylene wear with isolated liner exchange in a well-fixed acetabular cup has become an increasingly common procedure in revision THA [7]. Changing the PE liner can limit morbidity and avoids bone loss associated with removal of a well-fixed cup [7, 19]. The literature reports that the implant survival rate after liner replacement is comparable with that observed after more complex revisions, but that the risk of THA dislocation is significantly higher [19].

After revision total hip arthroplasty (rTHA), dislocation rates of up to 39% are reported [20] as compared to incidences ranging from 0.5% to 5% after primary THA [12, 21, 22]. The source of this increase in dislocations after revision surgery is still not fully understood and appears to be multifactorial [23]. In our patient series the dislocation rate after PE liner exchange for wear was 15.8%. This rate is consistent and well in line with previously published studies regarding rTHA because of PE wear [1, 5].

Several studies have shown that larger femoral head size directly influences stability [24, 25]. A recent study by Faldini et al. mentioned that it is advisable to use a 36-mm head diameter or larger when performing rTHA [23]. In our study, the head size most commonly used for revision surgery was 32 mm in both groups. Unfortunately, there was insufficient data to analyze our results on head size.

To reduce the risk of dislocation after rTHA some authors advocate using an elevated rim liner; even though a poorly positioned and elevated rim liner can inadvertently result in impingement with the iliopsoas muscle or tendon, leading to anterior hip pain [24, 26]. In addition, the study by Labek et al. demonstrated a very high failure rate for constrained liners [27]. With regard to dislocation rate, we found no advantage in using a 10° lipped or constrained liner, but there was insufficient data to analyze our results considering the above-mentioned liners. Further investigation is needed.

The effect of age on the dislocation rate after rTHA is still the subject of controversy. Wetters et al. [25] found that younger patients have a greater risk of dislocation after rTHA. Jo et al. [28] found dislocation rates to have no significant age-dependency. Yoshimoto et al., on the other hand, identified advanced age as a significant independent risk factor for dislocation. In our study, the two groups showed no significant differences in age composition or comorbidity index. We, therefore, detected no influence of age or comorbidity on the probability of dislocation.

In a study by Kosashvili et al. dislocation rates in patients undergoing first-time revisions were found to be significantly lower than in those undergoing repeat revisions [24, 28–31]. Some authors suggest combining revision of the femoral and the acetabular side to achieve the best positioning and restoration of correct offset [24, 32]. A previously published study found dislocation rates for acetabulum-only revisions to be significantly higher than those for both components and femur-only reconstructions within the group of first-time revisions [24]. According to the literature, a reduction in rTHA dislocation can be achieved using dual-mobility cups [33–37]. The risk of dislocation seems to increase with every additional surgical procedure. In our study, we found dislocations when using lipped or constrained liners and when enlarging the head size. We found no difference related to the choice of surgical approach for primary THA of rTHA. Nevertheless, in 7.3% of our study population a revision of the cup in the observed follow-up period became necessary because of recurrent dislocations after liner exchange. Unfortunately, our data are too insufficient to show a statistically significant difference. Neverhteless, a trend can be seen and further investigation is needed.

To the best of our knowledge, we are the first study to date to investigate dislocation rate after liner exchange using EBRA measurements for wear and cup migration. A recently published study by Abrahams et al. reported accuracy of the EBRA cup in uncemented acetabular components [38]. In addition, for EBRA cup and radiostereometric analysis (RSA) the authors reported good agreement on classification of components that migrated proximally 1 mm up or down at two years with 100% sensitivity and 87% specificity [38]. In our study, EBRA analysis showed significantly greater medial and cranial cup migration in the study group. Secondary instability might have occurred after initial subsidence. This may be understood as an early sign of later aseptic loosening and a symptom of osteolytic weakening of the bone stock [39], but this investigation was not part of the present study.

Another very important issue for recurrent dislocations in patients with wear of the polyethylene liner is seen to be the pathophysiological mechanisms of PE wear. In recent studies the pathophysiological mechanisms of PE wear and PE wear-induced osteolysis have been studied extensively [40–42]. It has been shown that debris particles can induce a cellular response in periprosthetic tissues, with the up-regulation of toll-like receptors (TLRs) on macrophages [43, 44]. TLR signaling leads to up-regulation of many chemokines and cytokines, such as TNF-α, IL-1β, MCP1 and others [43, 44]. The inflammatory response that ensues leads to activation of osteoclasts and induction of local bone resorption [43, 44]. Lachiewicz, Watters and Oral et al. showed improved wear resistance for new generation polyethylene, as highly X-linked polyethylene and vitamin E-doped polyethylene by comparison with conventional liners [43, 44]. Although the description above was not underlying our study results, the wear rate in the study group was twice as high as compared with that of the control group. This could be a weak indication that the PE particle concentration in the surrounding soft tissue have been higher and, therefore, weaken the tightness of the collagen fibers. Nevertheless, our study revealed no significant difference in PE wear in either group. Even though we found no statistical significance with regard to wear rate, but our study may provides further insights into potential risk factors and might add information to the recent debate on dislocation after rTHA, especially concerning cup migration.

The present study has several limitations, such as the retrospective methodology and the selection bias of the study group. Patient follow-up was not blinded or randomized, for which reason bias and confounders are difficult to rule out. Furthermore, we concentrated on measuring cup positioning with EBRA, but the technology does not allow femoral component rotation to be measured to calculate combined anteversion as an important risk factor for prosthetic impingement and dislocation. A preoperative fluoroscopy-guided joint fluid sample was gathered to rule out low-grade infection. Unfortunately, no further investigation was performed, e.g., histopathological diagnosis of the synovia for identification of particles of prosthesis material or polyethylene particles. This would have been of great importance in evaluating implant failure. In addition, we didn't perform a power analysis wether the number of patients has enough volume to compare; instead we calculated a numerative factor with increased risk in percent and the number needed to harm.

Our study shows that exchange of the PE liner for wear entails a high risk of dislocation and an unexpectedly high risk of cup revision. In 46% of our study group patients the cup was revised. It would seem that this risk is increased when PE wear is combined with cup migration, thus potentially leading to instability. Surgeons should address individual risks, e.g., using large head diameters, dual-mobility cups, liners with elevated rim, and should stick to exact positioning, especially in patients with abductor deficiency. Further investigations are needed.

## Conclusion

In conclusion, isolated exchange of the polyethylene liner because of wear showed a high risk of dislocation and further cup revision. Further investigations are needed to assess if the threshold for revising well-fixed components in the case of liner wear should be lowered.

## Data Availability

Data will be sent if necessary.
